# Assessing the impacts of women’s autonomy on their approval of intimate partner violence: a nationwide cross-sectional study

**DOI:** 10.1186/s12889-025-24428-y

**Published:** 2025-09-24

**Authors:** Mahnaz Ibrahim, Mohammad Hridoy Patwary, Anisuddin Ahmed

**Affiliations:** 1 Institute of Statistical Research and Training (ISRT), University of Dhaka, Dhaka, Bangladesh; 2 Maternal and Child Health Division, International Centre for Diarrhoeal Disease Research, Dhaka, Bangladesh; 3 Global Health and Migration Unit, Department of Women’s and Children’s Health, Uppsala University, Akademiska Sjukhuset, Uppsala, 751 85 Sweden

**Keywords:** Intimate partner violence, Women’s autonomy, BDHS, Multivariable binary logistic regression.

## Abstract

**Background:**

Intimate partner violence (IPV) is a severe issue in many low-and middle-income countries and remains a persistent public health problem. Research suggests that controlling attitudes by husbands can increase the risk of women experiencing IPV. To delve deeper into this issue, this study aims to investigate the relationship between women’s autonomy and their approval of IPV against women.

**Methods:**

This study utilized the Bangladesh Demographic and Health Survey (BDHS) 2017-18, a cross-sectional nationally representative survey consisting of 20,127 ever-married women aged 15–49. Both unadjusted and adjusted associations between acceptance of IPV against women and four dimensions of autonomy— economic decision-making, self-health-related decision-making, freedom of movement, and non-threatening sexual agreement within partnerships—were investigated. Chi-square tests were used to assess bivariate associations, followed by Cramer’s V to measure the strength of these associations. A multivariable binary logistic regression model was then applied to estimate adjusted associations.

**Results:**

One in every five women approved of IPV. Among the four dimensions of women’s autonomy, women having autonomy in self-health care had the lowest prevalence in accepting IPV (6.52%). Except for self-economic decision autonomy, the other three dimensions of women’s autonomy showed a significant unadjusted or preliminary association with the perception of approving IPV (p-value < 0.01). Women who shared control over their earnings with their husbands, rather than exercising solo control, demonstrated higher odds of approving IPV (AOR 1.16, p-value 0.04). Additionally, women lacking the final say on important household purchases and visits to close relatives exhibited increased odds of approving IPV compared to those with sole decision-making power (AOR 1.35, p-value < 0.001; AOR 1.37, p-value < 0.001, respectively). Conversely, women who lacked the final say on their own health care were less likely to approve of IPV compared to those with sole decision-making authority (AOR 0.60, p-value < 0.001).

**Conclusion:**

This study highlighted that a significant number of women in Bangladesh approve of violence by their husbands, which could be a major obstacle to the reduction of violence in society. Further, the results indicate a significant correlation between women’s autonomy—in terms of decision-making, mobility, freedom from threatening sexual relations with their husbands, and access to and control over economic resources— and their approval of IPV. Strengthening women’s autonomy emerges as a vital strategy for decreasing the acceptance of IPV, advancing gender equality, and enhancing the overall well-being of women.

**Supplementary Information:**

The online version contains supplementary material available at 10.1186/s12889-025-24428-y.

## Introduction

### Background of the study

Intimate partner violence (IPV) is a highly prevalent form of gender-based violence, denoting a spectrum of abusive and coercive actions within intimate relationships, including physical, sexual, and psychological attacks [1–3]. The World Health Organization (WHO) defines IPV as any behavior in an intimate relationship that causes psychological, physical, or sexual harm, including physical aggression, controlling behavior, psychological abuse, and economic and sexual coercion [4]. In the context of Bangladesh, the physical aspect of violence, particularly wife-beating, is more relevant and critical to understanding the issue of IPV. The impacts of IPV are profound, resulting in both short-term and long-term adverse effects on women and their children [5]. Victims may suffer from various physical and psychological harms, including fractures, lacerations, traumatic brain injuries, memory loss, gynecological disorders, chronic pain syndromes, depression, post-traumatic stress disorder (PTSD), and suicidal thoughts or attempts [6–11]. Even after the abuse has ceased, IPV survivors may still suffer from physical health repercussions, with abused women having a 50–70% increase in gynecological, central nervous system, and stress-related problems [12].

Despite the detrimental effects, the approval of IPV dominantly prevails in low and middle-income countries [13]. One of the most dreadful features of IPV is that it is often socially accepted [14]. The approval of IPV is often rooted in patriarchal norms that reinforce male dominance and control over women. These norms are continued through cultural narratives that depict violence as a legitimate means of asserting authority within intimate relationships [15]. Such ideologies not only harm individual victims but also contribute to a societal framework that allows IPV to persist unchallenged. The normalization of violence against women can lead to increased rates of IPV, as seen in various studies that link societal attitudes with the prevalence of domestic violence [13, 16] The IPV approval and victim-blaming not only affects the immediate victims but also has broader societal implications. It fosters an environment where violence against women is tolerated and even normalized, making women more vulnerable to the abuse. According to 48 population-based surveys conducted worldwide, a significant percentage of women, ranging from 10 to 70%, have reported being physically harmed by their male partners at some point in their lives [17]. The act of violence against women by their partners remains elevated and varies across regions [18, 19]. In the early 2000s, studies indicated that approximately 60% of women reported experiencing physical IPV in their lifetime in Bangladesh, a figure that has remained relatively consistent in subsequent surveys, such as the 2016 Bangladesh Demographic and Health Survey, which reported that about 50% of women aged 15–49 experienced physical violence from their husbands [20]. This suggests that the actual rates of IPV have not seen a substantial decline.

Approval of IPV is more common in Africa and South Asia, and less common in Central and Eastern Europe, Latin America, and the Caribbean [13]. This could be due to the cultural and social norm differences in various regions. In Bangladesh, which makes up 9.2% of South Asia’s female population, 69% of women have experienced physical or sexual abuse by their husbands, leading to serious mental and physical health problems [23–27]. According to the Bangladesh Bureau of Statistics (BBS) 2015, half of the country’s women experience physical violence perpetrated by their husbands during their lifetime. However, many women continue to support this human rights violation, which perpetuates IPV and reflects lower self-esteem among these individuals [21–23]. Therefore, it is crucial to understand why some women approve of IPV, as this insight can help explain the dynamics of perpetration, victimization, and responses to such behavior.

Women’s autonomy refers to their ability to influence decisions that affect themselves and their immediate family, control economic and information resources, and move around freely, which plays a critical role in the social structure of modern society and is vital for social development [24]. Women’s autonomy carries a favorable impact on socio-economic and demographic aspects of a nation, including market involvement, contraceptive use, fertility, and greater child welfare [24–26]. Additionally, the decision-making authority correlated with reduced rates of unintended pregnancies, as women could make choices about contraception and family planning [27]. The higher control for self-health decision-making implications provided the opportunity to seek the proper utilization of maternal healthcare, which is essential for enhancing maternal health and child welfare [27–29]. In the social settings of Bangladesh, autonomous women could be at lower risk of experiencing IPV due to their increased decision-making power and access to resources enabling them to walk out of an abusive relationship and equipping them to negotiate safe relationships and resist coercive behaviors [30].

### Rationale and objectives of the study

In the patriarchal context of Bangladesh, most women typically rely on financial support and social security, leading to a lack of their decision-making implications and greater tolerance for IPV [31, 32]. Women who view IPV as acceptable or normative are more prone to self-blame, often internalizing the violence as a reflection of their own shortcomings. This mindset can lead to long-term mental health issues, such as depression and anxiety. Additionally, these women are less likely to seek help or report the violence to civil authorities or family members, further isolating themselves and perpetuating the cycle of abuse [33]. While numerous studies have explored IPV, women’s autonomy, and their effects on various health outcomes in Bangladesh, there remains a gap in understanding how women’s autonomy influences their approval of IPV. Existing literature on IPV in Bangladesh often overlooks the influence of women’s autonomy on their attitudes towards IPV [34–39]. To address this existing knowledge gap, the current study investigated the impact of women’s autonomy on their approval of IPV in Bangladesh. This should contribute to a primary concern of the United Nations’ Sustainable Development Goals (SDGs), particularly goal 5.1 (end all forms of discrimination against all women and girls everywhere) and goal 5.2 (eliminate all forms of violence against all women and girls) [40] by helping to design intervention strategies. Specifically, the study aimed: (a) to explore the prevalence of IPV acceptance among different demographic and age groups, (b) to evaluate the sociodemographic factors that drive IPV approval, and (c) to estimate the influence of women’s autonomy on their approval of IPV.

### Theoretical framework

The *social learning theory* highlighted that individuals learn behaviors through observation and imitation. People exposed to environments where IPV is normalized or approved of would be more likely to approve of it in their own relationships [41]. Thus, higher acceptance of IPV could be an indication of the existing troubling situation. The historical patterns of IPV in Bangladesh emphasized alarmingly high prevalence [20, 30]. Due to the cultural norms in Bangladesh, women observe or learn that men are approved and even positively reinforced for using violence against women to enforce discipline and are likely to internalize these accepting attitudes. Research has demonstrated that women who adopt such beliefs often internalize the notion that men have the right to physically discipline women when they deviate from prescribed gender roles [42, 43]. Hence, Bangladeshi women are prone to give up their rights or condition of self-government to avoid the abuse. This socialization process reinforces harmful norms and perpetuates a cycle of violence, making it essential to address these attitudes in efforts to combat IPV.

The *feminist theories* provided a theoretical framework for comprehending wives’ submissive and husbands’ controlling behaviors and the driving force for IPV-related practices and the attitudes toward it [44, 45]. The propositions of the theories stated that gender could be a form of structural inequality woven into social relationships, primarily creating vulnerability for women in relation to men’s control. In social interactions, gender roles dictate men to take part in activities that portray them as authoritative and enhance their ability to impede women’s autonomy [46]. The power inequalities to exercise decisions tend to increase the likelihood of wife beating, specifically in families dominated by husbands [47, 48]. These findings indicate that women with no autonomy for self-government, are at higher risk of reporting the experience of IPV and approving it. The current study is specifically grounded in *intersectional feminism -* a strand of feminist theory, which emphasizes how overlapping social identities such as gender, socioeconomic status and cultural norms shape women’s lived experiences and access to power [49]. This theoretical framing encouraged the study to hypothesize that examining women’s autonomy, in terms of decision-making, mobility, freedom from threatening sexual relations with their husbands, and access to and control over economic resources could provide valuable insights into their approval of IPV. Accordingly, it was posited that higher autonomy across these key domains is associated with decreased approval of IPV.

Exploring the interplay between women’s autonomy and their approval of IPV would help to design comprehensive intervention programs to fight the unfounded beliefs, enhance women’s decision-making abilities, reduce IPV victimization, and thereby improve the overall well-being of women in Bangladesh. The covariates in such studies relating to attitudes toward violence and women’s autonomy are selected through applied knowledge and literature review. Initially, the current study listed the probable covariates based on the literature. Further, following the definitions of the systemic levels of social framework given in the nested ecological theory by Dutton (2006), a comprehensive literature review, and the framework of *intersectional feminism*, the final list was created. Supplementary Table 1 shows the sociodemographic variables of the study at the systematic levels.

## Methods

### Data overview

This study utilized data from the 2017-18 Bangladesh Demographic and Health Survey (BDHS), which is the eighth nationwide assessment conducted by the National Institute of Population Research and Training (NIPORT) in collaboration with the Demographic and Health Surveys (DHS) program. The objective of this survey was to assess the demographic and health status of women and children in Bangladesh and provide estimates for 14 major indicators of the Health Population and Nutrition Sector Program (HPNSP) Results Framework (Ministry of Health and Family Welfare 2017). For this purpose, the survey adopted a two-stage stratified sampling approach. In the first stage of the survey, 675 enumeration areas (EAs) were chosen as primary sampling units (PSUs), selected with a probability proportional to EA size. These EAs comprised 250 urban areas and 425 rural areas. In the second stage of the survey, a systematic sampling method was employed to collect data from approximately 30 households per EA. Sampling weights were applied to estimate indicators representative at both the national and divisional levels. The current study used information on 20,127 ever-married women aged 15–49. The authors were given access to the dataset for research purposes on February 9, 2022. Ethics approval and consent to participate. The DHS program was reviewed and approved by the Institutional Review Board (IRB) of ICF International, Rockville, Maryland, USA. There were multitudes of surveys implemented under this program, and the data from the latest survey, 2017-18 Bangladesh DHS, was used in this study. The 2017–18 Bangladesh DHS was also approved by another ethical committee: The Bangladesh Medical Research Council. This BDHS was conducted under the authority of the National Institute of Population Research and Training (NIPORT) of the Government of the People’s Republic of Bangladesh, with financial support from USAID/Bangladesh. The IRB-approved procedures for DHS public-use datasets do not in any way allow respondents to be identified. An interview was conducted only if the respondent provided their verbal consent in response to being read an informed consent statement by the interviewer. Those who refused to consent were excluded from the survey.

### Dependent variable

 The outcome variable for this study was approval of IPV. The BDHS 2017-18 had five questions regarding the perception of the respondent on IPV against women, which included the respondent stating that she approved of IPV in cases of a wife was doing the following: (1) “going out without telling the husband”, (2) “neglecting the children”, (3) “arguing with the husband”, (4) “accidentally burning food”, (5) “refusing to have sex”. Based on the DHS guidelines and prior studies [50], these five binary indicators were combined into a single binary variable called approval of IPV, with two possible responses - yes or no. If the woman answered “no” to all of these five questions, the response was considered “no” for the IPV approval variable. Otherwise, it was considered “yes”. No indicated a score of “0” and yes indicated “1”, making the outcome range from 0 to 5, which was combined into two categories: “0” indicating the respondent” did not approve of IPV,” and 1–5 were coded as “1” implying the respondent “approved of IPV.” This standardized set of questions and the resulting binary indicator is commonly used in DHS surveys to measure attitudes towards IPV [51].

### Independent variables

 Supplementary Table 1 contains all the independent variables that were considered in this study. The study focused on the four dimensions of women’s autonomy of interest: economic decision-making, self-health-related decision-making, freedom of movement, and non-threatening sexual agreement with the husband. According to DHS guidelines, the economic decision-making autonomy dimension is defined as who usually decides how to spend the respondent’s earnings. The self-health-related decision-making dimension is characterized as decisions regarding respondents’ health-related decisions. Freedom of movement autonomy is characterized as someone who usually has a say on visits family or relatives. Lastly, the sexual autonomy dimension is identified as if the respondent can negotiate sexual relations and refuse sex with the husband.

To assess women’s autonomy in various domains, we categorized the decision-making variables related to control over earnings, final say on health-care decisions, ability to visit family or relatives, and authority over large household purchases. Each of these variables originally consisted of five categories—(a) “respondent alone,” (b) “respondent and husband jointly,” (c) “husband alone,” (d) “father/mother-in-law,” and (e) “others.” Since our primary concern is to understand whether a woman has full control, is partially involved, or has no involvement in these critical decisions, all variables were recoded into three categories: (a) “respondent alone” indicating complete autonomy, (b) “respondent and husband jointly” indicating shared decision-making, and (c) “others” encompassing all scenarios where the respondent lacks sole or joint decision-making authority.

Ability to negotiate sexual relations with husband: Being able to deny a husband’s advances underlines a woman’s sexual liberty. In the original dataset, the variable of wife can refuse to have sex has three categories: (a) “no”, (b) “yes”, and (c) “do not know/ depends”, which was categorized into two groups: (a) “no” and (b) “yes” characterizing whether a woman has the power to refuse sex and classifying do not know or depends as missing data. Ability to ask husband to use condom: Having control over birth control methods may suggest control over one’s health care and the ability to negotiate sexual interactions with husband. In the original dataset, the variable of wife can refuse to have sex has three categories: (a) “no”, (b) “yes”, and (c) “do not know/ depends”, which was categorized into two groups: (a) “no” and (b) “yes” determining if a woman has the right to obtain that her spouse wears protection and classifying they do not know or depends as a missing category. Other independent variables are division, place of residence, respondent’s education level, husband’s education level, household wealth index, religion, no. of living children, and respondent’s age (in years).

### Statistical analyses

To ensure the accurate representation of the study population and unbiased standard errors provided by the models, the data was adjusted for the individual weights and strata/cluster variations. Univariate analysis was employed to assess the distribution of the sociodemographic variables used in the analysis. The percentage distribution of approval of IPV over different factors was calculated. The association between the study factors and approval of IPV was tested using the chi-squared test and Cramer’s V was calculated to assess the strength of these associations. A spatial distribution of approval of IPV against women was also used to assess the division-level pattern. Further, the primary outcome of interest, approval of IPV, was modeled using a generalized linear model (GLM) with a logit link function to estimate the adjusted effects of the study factors. The calculated adjusted effects (AORs) along with their statistical significance and 95% confidence intervals were provided. Throughout the study, a statistical significance level of 5% was considered. All analyses were conducted using Stata V.14.0 (Stata SE V.14, Stata Corp).

## Results

### Descriptive analysis

#### Univariate analysis

Table 1 represents the distribution of women aged 15–49 in Bangladesh by their sociodemographic characteristics. Among those who enjoyed economic decision-making autonomy, a large proportion had to share it with their husbands (59.57%) compared to claiming it alone (32.39%). Only 9.72% of women had complete control over their health-care choices. Most of those with healthcare decision-making autonomy had to consult with their husbands (66.72%). Compared to the previous two decision-makings, authority over own household major purchases was even bleaker. Only 4.86% of women could make this choice on their own, with the majority of them consulting their spouses (66.91%). Women who had a say in visits to family or relatives face the same situation. Nearly two-thirds of those involved in the decision-making process conferred with their husbands, and 8.42% had exclusive authority. Regarding sexual agreements with spouses, most women (86.09%) could refuse to have sex with their husbands and ask for protection (80.00%).

**Table 1:** Sociodemographic characteristics of the study participants

Women’s autonomy indicators	*n* (%)
Control over respondent’s earnings	
Respondent alone	2513 (32.39)
Respondent and husband jointly	4622 (59.57)
Others	624 (8.04)
Final say on respondent’s health-care	
Respondent alone	1845 (9.72)
Respondent and husband jointly	13,000 (66.72)
Others	4472 (23.56)
Final say on making large household purchases	
Respondent alone	923 (4.86)
Respondent and husband jointly	13,000 (66.91)
Others	5358 (28.23)
Final say on visits to family or relatives	
Respondent alone	1598 (8.42)
Respondent and husband jointly	13,000 (66.09)
Others	4840 (25.50)
Final say on husband’s earnings	
Respondent alone	764 (4.02)
Respondent and husband jointly	12,000 (64.50)
Others	5637 (29.69)
Ability to negotiate sexual relations with hus- band	
Yes	16,000 (86.09)
No	2641 (13.91)
Ability to ask partner to use condom	
Yes	15,000 (80.00)
No	3797 (20.00)
Sociodemographic variables	
Place of residence	
Urban	5729 (28.46)
Rural	14,000 (71.54)
Division	
Dhaka	5123 (25.46)
Chittagong	3622 (17.99)
Barisal	1125 (5.59)
Khulna	2336 (11.61)
Mymensingh	1546 (7.68)
Rajshahi	2802 (13.92)
Rangpur	2380 (11.83)
Sylhet	1192 (5.92)
Wealth index	3743 (18.60)
Poorest	
Poorer	3957 (19.66)
Middle	4059 (20.17)
Richer	4184 (20.79)
Richest	4184 (20.79)
Education	
No education	3333 (16.56)
Primary	6290 (31.25)
Secondary	7974 (39.62)
Higher	2530 (12.57)
Husband’s education	
No education	4078 (21.54)
Primary	6080 (32.12)
Secondary	5675 (29.98)
Higher	3098 (16.36)
Religion	
Islam	18,000 (90.68)
Others	1877 (9.32)
No. of living children	
0	3126 (15.53)
1–2	13,000 (63.98)
3–4	3791 (18.84)
5+	332 (1.65)
Respondent’s age in years (mean (SD))	31.59 (9.18)

The two southern divisions, Dhaka and Chittagong, had the most significant percentage of women of the eight divisions. Slightly below three-quarters of the women in the survey resided in rural areas. The women in the study were, on average, 31.59 years old. Distribution was almost homogeneous across the wealth quintile groups. Over half and slightly less than two-thirds of the women (63%) had 1–2 children, whereas less than one-fifth (1.65%) had five or more children. Of those who had a say on how to spend their husbands’ earnings, the majority (64.50%) shared this decision-making with their husbands, and about 4% decided by themselves. Those who had a voice in how their husband’s earnings were divided into two groups: those who shared the choice with their spouses (64.50%) and those who made the decision alone (4%). As expected, most of the women in the survey followed Islam as a religion, while a small percentage of women followed other predominant religions. Only one-quarter of the women had attained higher education, and 21.54% had no formal education.

### Bivariate analysis

Table 2 presents the percentages of women who approved IPV by the key sociodemographic and autonomy dimension variables addressed in the study through bivariate analyses. Overall, 20.11% of women were found to approve of IPV. Results showed that economic decision-making initially had a homogeneous distribution of women approving IPV. A higher prevalence of approving IPV was evident when a woman lost her health-care decision-making control from having complete control over it (6.52%) to sharing the control with her spouse (18.64%) and not having it (22.49%).

**Fig. 1:**
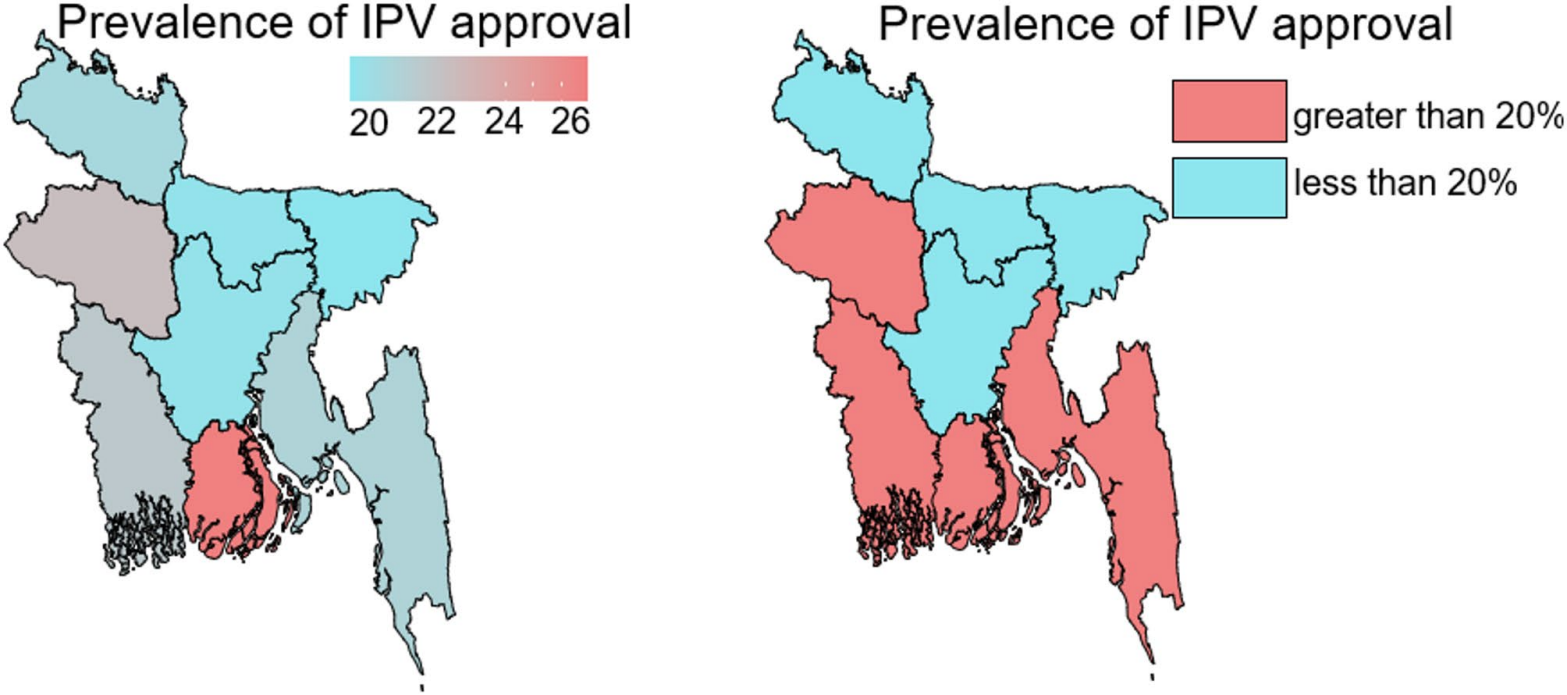
Division-level spatial distribution of IPV approval

When decision-making was shared with husband jointly, there was a lower prevalence of women accepting IPV for final say on large household purchases and final say on visiting family or relative variables (18.61% and 18.15%, respectively), compared to having complete control or no control over the decision-making procedure. The approval of IPV is lower when women can negotiate sexual relations with their husbands (18.44%) than when they are not able to negotiate sexual relations with husbands (31.63%). When women couldn’t ask their partners to use protection, there was a higher proportion of approval of IPV (28.19%) than when they could (18.29%). The percentage of women approving IPV was lower in the north-eastern divisions of Bangladesh compared to the national average of 20% (Fig. 1). The prevalence of IPV acceptance had decreased as respondents’ education levels had increased. The highest degree of approval for IPV was seen among women with no education (25.75%), and it reduced to 23.81% among women with primary education. As the education degree progressed from primary to secondary, the percentage dropped to 18.42%. The lowest prevalence of approval to IPV was seen among women with higher education levels (8.83%). As the respondent’s husband’s educational level increased, the prevalence of IPV acceptance decreased. The highest degree of approval for IPV was seen among women when husbands had no education (26.21%), and it reduced to 23.42% among women when their husbands had attained primary education. As the education degree progressed from primary to secondary, the percentage fell to 17.7%. The lowest prevalence of approval for IPV was seen among women when their husbands had a higher education level (11.6%). Women who accept IPV were, on average, 32.09 years old.

**Table 2:** The results of bivariate analysis representing association between determinants and approval for IPV with the p-value

Variables	Total women (*N*)	% Women approving IPV	Cramer’s V			*p*-value	
Women’s autonomy indicators							
Overall	20,127	20.11					
Control over respondent’s earnings							
Respondent alone	2513	20.44					
Respondent and husband jointly	4622	20.53	0.04			0.62	
Others	624	22.49					
Final say on respondent’s health-care							
Respondent alone	1845	6.52					
Respondent and husband jointly	13,000	18.64	0.05			< 0.01	
Others	4472	22.33					
Final say on making large household purchases							
Respondent alone	923	24.61					
Respondent and husband jointly	13,000	18.61	0.06			< 0.01	
Others	5358	23.46					
Final say on visits to family or relatives							
Respondent alone	1598	24.66					
Respondent and husband jointly	13,000	18.15	0.07			< 0.01	
Others	4840	24.31					
Final say on husband’s earnings							
Respondent alone	764	25.1					
Respondent and husband jointly	12,000	18.73					
Others	5637	23.19	0.04			< 0.01	
Husband has no earnings	339	16.55					
Ability to negotiate sexual relations with husband							
Yes	16,000	18.44					
No	2641	31.63	0.12			< 0.01	
Ability to ask partner to use condom							
Yes	15,000	18.29	0.10				
No	3797	28.19				< 0.01	
Sociodemographic variables							
Place of residence							
Urban	5729	16.69					
Rural	14,000	21.47	0.03			< 0.01	
Division							
Dhaka	5123	18.66					
Chittagong	3622	20.01					
Barisal	1125	26.59					
Khulna	2336	20.88					
Mymensingh	1546	18.6	0.11			0.17	
Rajshahi	2802	21.77					
Rangpur	2380	19.54					
Sylhet	1192	18.38					
Wealth index							
Poorest	3743	24.55					
Poorer	3957	22.4					
Middle	4059	20.93	0.09			< 0.01	
Richer	4184	19.27					
Richest	4184	14.02					
Respondent’s education level							
No education	3333	25.75					
Primary	6290	23.81	0.13			< 0.01	
Secondary	7974	18.42					
Higher	2530	8.83					
Husband’s education level	4078						
No education		26.21					
Primary	6080	23.42	0.11			< 0.01	
Secondary	5675	17.7					
Higher	3098	11.16					
Religion							
Islam	18,000	20.78	0.05				
Others	1877	13.59					
No. of children	3126					< 0.01	
0		17.88	0.04				
1–2	13,000	19.81					
3–4	3791	22.48					
5+	332	25.97					
Respondent’s age in years (mean (SD))	31.59 (9.18)	32.09 (9.32)				< 0.01	

### The multivariable binary logistic regression model

From Table 3, several sociodemographic factors were significantly associated with the approval of IPV, including decision-making on healthcare, ability to negotiate sexual relations, ability to ask the partner to use a condom, division, respondent’s education level, husband’s education level, religion, and respondent’s age. Control over respondent’s earnings is significantly associated with approval to IPV, indicated by 16% (AOR: 1.16, p-value: 0.040) greater odds of accepting IPV for those women sharing the economic decision-making with husband compared to those the odds of those who had complete economic decision-making autonomy. The final say on the respondent’s health care is significantly associated with the approval for IPV, indicated by 37% (AOR: 0.63, p-value: <0.001) lower odds of accepting IPV when the wife and husband jointly.

**Table 3:** Results from the generalized linear model fitted with the approval of IPV to sociodemographic variables showing adjusted odds ratios (AORs), 95% confidence interval, and p-value

Variables	AORs (95% CI)	*p*-value
Women’s autonomy indicators		
Control over respondent’s earnings		
Respondent alone (Ref.)		
Respondent and husband jointly	1.16 (1.00, 1.33)	0.04
Others	0.95 (0.76, 1.20)	0.70
Final say on respondent’s health-care		
Respondent alone (Ref.)		
Respondent and husband jointly	0.63 (0.50, 0.78)	< 0.001
Others	0.60 (0.47, 0.77)	< 0.001
Final say on making large household purchases		
Respondent alone (Ref.)		
Respondent and husband jointly	1.11 (0.81, 1.53)	0.5
Others	1.35 (0.97, 1.88)	0.008
Final say on visits to family or relatives		
Respondent alone (Ref.)		
Respondent and husband jointly	1.04 (0.81, 1.33)	0.81
Others	1.37 (1.05, 1.80)	0.02
Final say on husband’s earnings		
Respondent alone (Ref.)		
Respondent and husband jointly	0.86 (0.63, 1.18)	0.34
Others	0.93 (0.68, 1.28)	0.67
Husband has no earnings	0.69 (0.40, 1.19)	0.18
Ability to negotiate sexual relations with husband		
No (Ref.)		
Yes	0.61 (0.52, 0.71)	< 0.001
Ability to ask partner to use condom		
No (Ref.)		
Yes	0.79 (0.68, 0.91)	< 0.001
Sociodemographic variables		
Place of residence		
Urban (Ref.)		
Rural	0.99 (0.85, 1.17)	0.98
Division		
Dhaka (Ref.)		
Chittagong	1.13 (0.92, 1.40)	0.24
Barisal	2.30 (1.77, 2.96)	< 0.001
Khulna	1.30 (1.06, 1.61)	0.01
Mymensingh	0.88 (0.67, 1.13)	0.33
Rajshahi	1.20 (0.85, 1.30)	0.07
Rangpur	1.05 (0.85, 1.30)	0.62
Sylhet	0.95 (0.68, 1.33)	0.76
Wealth index		
Poorest (Ref.)		
Poorer	0.91 (0.77, 1.07)	0.26
Middle	0.89 (0.75, 1.07)	0.23
Richer	0.92 (0.76, 1.12)	0.45
Richest	0.87 (0.68, 1.12)	0.29
Respondent’s education level		
No education (Ref.)		
Primary	0.90 (0.77, 1.06)	0.24
Secondary	0.68 (0.56, 0.83)	< 0.001
Higher	0.45 (0.32, 0.62)	< 0.001
Husband’s education level		
No education (Ref.)		
Primary	0.97 (0.84, 1.13)	0.76
Secondary	0.91 (0.93, 1.40)	0.3
Higher	0.72 (0.85, 1.96)	0.003
Religion		
Islam (Ref.)		
Others	0.72 (0.59, 0.89)	0.002
No. of children		
0 (Ref.)		
1–2	1.08 (0.90, 1.29)	0.40
3–4	1.13 (0.92, 1.40)	0.21
5+	1.29 (0.85, 1.96)	0.22
Respondent’s age in years	0.99 (0.98, 1.00)	0.05

### AOR: adjusted odds ratio, CI: confidence interval

decided the wife’s health care. Women who did not have freedom of movement had 37% (AOR: 1.37, p-value: 0.020) higher odds of approving IPV compared to those who had freedom of movement. Women who had sexual autonomy, such as those who could refuse sex and ask their husband to use protection, had 39% (AOR: 0.61, p-value: <0.001) and 21% (AOR: 0.79, p-value: <0.001) lower odds of approving IPV compared to the odds of those who did not have the autonomy. Among the eight divisions, women residing in Barisal and Chittagong, the odds of them approving IPV were 2.3 times (AOR: 2.30, p-value: <0.001) and 30% (AOR: 1.30, p-value: 0.010) higher than the odds of women residing Dhaka. Women who followed other religions had 28% (AOR: 0.72, p-value: 0.002) lower odds of approving IPV than the odds of Muslim women. All the autonomy dimensions of interest were found to have significant associations with the approval of IPV. Attaining secondary and higher education was associated with 32% (AOR: 0.68, p-value: <0.001) and 55% (AOR: 0.45, p-value: <0.001) lower odds of accepting IPV when compared to attaining no education. Husband with higher education was associated with a 28% (AOR: 0.72, p-value: 0.003) lower odds of approval of IPV.

## Discussion

This study explored the relationship between women’s autonomy and the approval of intimate partner violence among ever-married women in Bangladesh. Additionally, it has identified high-risk groups more inclined to adhere to this belief. The analysis revealed that economic, self-health-related decision making, freedom of movement, and sexual agreement with the husband were significantly associated with approval for IPV. Furthermore, the sociodemographic variables, such as the age of the women, their educational levels, the highest level of education attained by their husbands, residing in particular divisions, and their religion, were significantly associated with approval for IPV, aligning with the hypotheses grounded in intersectional feminist theory. The link between economic decision-making autonomy and lower odds of approving IPV can be explained by the fact that women with control over economic resources feel more empowered. This empowerment can lead to a decrease in acceptance of abusive behaviors. Economic autonomy often provides individuals with the means to leave abusive situations by providing financial independence. This can lead to more efficient use of resources by themselves, which may influence their perception of IPV [52]. Though our initial analysis did not find a significant association between economic decision-making and approval for IPV, further analysis showed that sharing decision-making with one’s husband increased the odds of approving IPV, compared to having complete autonomy. The increased odds of approval of IPV with self-health-related decision making, in the context of Bangladesh can be thought of as a complex interplay among fear of social stigmatization, cultural norms and gender roles. Women may fear social repercussions for asserting their health autonomy, leading them to accept IPV as a way to maintain family harmony and avoid community disapproval [14]. The study found that freedom of movement of women, decreased their odds of approving IPV. This result aligns with the findings of a study conducted among Bangladeshi women [30]. Limited movement often leads to isolation, making women more vulnerable to controlling behaviors and violence. When women can move freely, they are less likely to feel trapped in an abusive situation, thereby decreasing the approval of IPV. Women’s sexual autonomy, defined as the capacity to refuse sex and negotiate safe practices, is linked to a reduced risk of approving IPV. This aligns with findings by Tesema, who found a statistically significant association between sexual autonomy and lower IPV acceptance prevalence across multiple sub-Saharan African countries [53]. Women who exercise sexual autonomy often may have more control over their reproductive health and choices, which could reduce their dependency on male partners. This independence makes it less likely that they will tolerate or approve IPV as a means of control. Consistent with the literature, the role of education, particularly the education level of both the respondent and her husband, emerged as a critical determinant in IPV acceptance [34]. Women with higher education levels and those married to educated husbands were less likely to approve IPV. Education fosters critical thinking and an awareness of human rights, which can significantly diminish the acceptance of violence among women. Educated women are more likely to understand the legal ramifications of IPV. This heightened awareness challenges traditional gender roles and promotes attitudes of equality within marital relationships, ultimately contributing to the rejection of violence as a societal norm [34, 54, 55]. Moreover, education often correlates with a higher socioeconomic status, enabling women to contribute financially to their families [56]. Socioeconomic status further influenced acceptance, as lower wealth quintile group was linked to higher approval rates of IPV [30]. Economic hardship can increase stress and conflict within households, potentially normalizing IPV as a coping mechanism [57]. Women in lower wealth quintiles may feel trapped in abusive relationships due to financial constraints and limited access to resources. Our study also found that women in rural areas were more likely to accept IPV, while urban women showed lower approval, which is consistent with previous studies [58–60]. This difference is likely due to stronger adherence to traditional norms, lower education levels, and greater economic dependence in rural regions. Urban women, with better access to education and resources, are more empowered to reject violence. The urban–rural difference may be the consequence of more remarkable socio-economic development in urban compared to rural areas [61]. Moreover, women from certain divisions were more likely to approve of IPV than others, potentially due to stronger traditional views on gender roles in these regions. Notably, Barishal and Chittagong divisions had higher approval rates. These divisional differences highlight the need for targeted interventions that address the specific cultural and economic factors that perpetuate IPV in these regions. Furthermore, age was a notable factor, with younger women tending to show higher acceptance of IPV. This finding aligns with previous research, which identified adolescent women as being at a higher risk for IPV than older women [62]. The marital status of women also affected their views on IPV; those who are currently married were more likely to accept it compared to unmarried women. Marital relationships come with societal expectations of loyalty and fidelity, which can lead women to rationalize IPV as a means of preserving familial stability. Study findings also revealed significant differences in IPV approval based on religious affiliation. Muslim women exhibited a higher tendency to approve IPV compared to their counterparts, which was consistent with previous literature [63, 64]. This disparity may stem from cultural beliefs within certain religious communities that affect attitudes towards gender roles and violence.

### Limitations

This study has several limitations. The cross-sectional nature of the data limits the ability to establish causality between women’s autonomy and approval of IPV. Longitudinal studies are needed to assess how changes in women’s autonomy over time influence attitudes toward IPV. Additionally, the reliance on self-reported data may introduce reporting bias, as women may under-report their approval of IPV due to social desirability concerns. Lastly, the decision to dichotomize the IPV approval variable; although useful for analytical clarity and consistency with previous studies, may lead to a loss of nuance and obscure variations in degrees of acceptance. Future studies should consider using mixed methods approaches to triangulate quantitative findings with qualitative insights into the contextual factors driving the approval of IPV.

## Conclusion

The current study provides critical insights into the protective role of women’s autonomy in reducing the approval of IPV in Bangladesh. Women with greater decision-making power, particularly in economic and visits to family or relatives house, were less likely to approve IPV. Additionally, the study identified high-risk groups with a greater likelihood of approving IPV, including women with limited education, low household wealth, rural residence, Muslim affiliation, older age, regional disparities, and husbands with lower educational attainment. Women who view IPV as acceptable are more likely to self-blame and avoid reporting the abuse. By focusing on this vulnerable population, policymakers can implement targeted initiatives that promote gender equality and support broader efforts to combat IPV in Bangladesh. Future research should further investigate the intersections between women’s autonomy and IPV approval to ensure interventions meet diverse socioeconomic and regional needs.

## Supplementary Information

Below is the link to the electronic supplementary material.

Supplementary Material 1

## Data Availability

The datasets generated and/or analyzed during the current study are available on the DHS program website, [https://dhsprogram.com/methodology/survey/survey-display-584.cfm](https:/dhsprogram.com/methodology/survey/survey-display-584.cfm).
