# Supplementary material for: Assessing the impacts of women’s autonomy on their approval of intimate partner violence: a nationwide cross-sectional study
Source: BMC Public Health. 2025 Sep 24;25:3075. doi: 10.1186/s12889-025-24428-y (PMC12462222; doi:10.1186/s12889-025-24428-y)
Supplement: Supplementary file 1 — Supplementary Material 1 [file 12889_2025_24428_MOESM1_ESM.docx]

**Assessing the Impacts of Women’s Autonomy on their Approval of Intimate Partner Violence: A Nationwide Cross-sectional Study**

Mahnaz Ibrahim^1*^, Mohammad Hridoy Patwary^1^

^1^Institute of Statistical Research and Training (ISRT), University of Dhaka, Dhaka, Bangladesh

**Supplementary Table 1.** Description of variables used in this study.

| **Sociodemographic factors** | **Description** |
| --- | --- |
| **Individual-level factors** |  |
| **Ability to negotiate sexual relationship with husband** | |
| Yes | Respondent can negotiate sexual relationship with husband. |
| No | Respondent cannot negotiate sexual relationship with husband. |
| **Ability to ask partner to use condom** | |
| Yes | Respondent has the ability to ask partner to use a condom. |
| No | Respondent does not have the ability to ask partner to use a condom. |
| **Control over respondent’s earnings** | |
| Controlled by respondent alone | Respondent has sole control over her earnings. |
| Controlled jointly by respondent and husband | Respondent’s earnings are jointly controlled with her husband. |
| Others | Other individual has control over respondent’s earnings. |
| **Final say on respondent’s health-care** | |
| Respondent alone | Respondent has the final say on health-care decisions. |
| Respondent and husband jointly | Self health-care decisions are made jointly by respondent and husband. |
| Others | Other individual has the final say on health-care decisions. |
| **Final say on making large household purchases** | |
| Respondent alone | Respondent has the final say on making large household purchases. |
| Respondent and husband jointly | Large household purchases are decided jointly with the husband. |
| Others | Respondent doesn’t have any say on large household purchases. |
| **Final say on visits to family or relatives** | |
| Respondent alone | Respondent has the final say on visits to family or relatives. |
| Respondent and husband jointly | Visits to family or relatives are decided jointly by respondent and husband. |
| Others | Other individual has final say on visits to family or relatives. |
| **Final say on husband’s earnings** | |
| Controlled by respondent alone | Respondent has sole control over husband’s earnings. |
| Controlled jointly by respondent and husband | Husband’s earnings are controlled jointly by respondent and husband. |
| Others | Respondent doesn’t have a final say on her husband’s earnings |
| **Respondent’s education level** | |
| No Education | Respondent has no formal education. |
| Primary | Respondent has 1-5 years of formal schooling. |
| Secondary | Respondent has 6-10 years of formal schooling. |
| Higher | Respondent has more than 10 years of formal schooling. |
| **No. of living children** |  |
| 0 | Respondent does not have any living children |
| 1-2 | Respondent have one to two living children |
| 3-4bl | Respondent have three to four living children |
| 5+ | Respondent have five or more living children |
| **Respondent’s Age** | Current age of the respondent in years |
| **Household-level factors** | |
| **Husband’s education level** | |
| No Education | Respondent’s husband has no formal education. |
| Primary | Respondent’s husband has 1-5 years of formal schooling. |
| Secondary | Respondent’s husband has 6-10 years of formal schooling. |
| Higher | Respondent’s husband has more than 10 years of formal schooling. |
| **Religion** |  |
| Islam | Religion of respondent is Islam. |
| Others | Religion of respondent is other than Islam. |
| **Wealth index** |  |
| Poorest | Respondent belongs to the poorest wealth index category. |
| Poorer | Respondent belongs to the poorer wealth index category. |
| Middle | Respondent belongs to the middle wealth index category. |
| Richer | Respondent belongs to the richer wealth index category. |
| Richest | Respondent belongs to the richest wealth index category. |
| **Community-level factors** |  |
| **Place of residence** |  |
| Urban | Respondent resides in an urban area. |
| Rural | Respondent resides in a rural area. |
| **Division** |  |
| Dhaka | Respondent belongs to the Dhaka division. |
| Chittagong | Respondent belongs to the Chittagong division. |
| Barisal | Respondent belongs to the Barisal division. |
| Khulna | Respondent belongs to the Khulna division. |
| Mymensingh | Respondent belongs to the Mymensingh division. |
| Rajshahi | Respondent belongs to the Rajshahi division. |
| Rangpur | Respondent belongs to the Rangpur division. |
| Sylhet | Respondent belongs to the Sylhet division. |
